# At the lower size limit for tetrapods, two new species of the miniaturized frog genus *Paedophryne* (Anura, Microhylidae)

**DOI:** 10.3897/zookeys.154.1963

**Published:** 2011-12-12

**Authors:** Fred Kraus

**Affiliations:** 1 Bishop Museum, 1525 Bernice St., Honolulu, Hawaii, USA

**Keywords:** clutch size, diminutive, ecomorph, Milne Bay Province, Mt. Dayman, Mt. Suckling

## Abstract

I describe two new species in the miniaturized microhylid frog genus *Paedophryne* from forests in southeastern Papua New Guinea. The first species is described on the basis of two specimens and exhibits female snout-vent length of 8.5–9.0 mm (no males known), whereas that of the second species, described on the basis of 12 specimens, is 8.8–9.3 mm, with males 8.1–8.9 mm. These frogs are smaller than the other two diminutive species described when the genus was recently erected, and they represent what are currently the smallest known species of tetrapods. The two species replace each other elevationally on the same mountain massif and occur in relative geographic proximity to the other named species of the genus. Females of both species contain only two enlarged ova, suggesting that they also possess clutch sizes at the extreme lower end of variation in frogs. All species of *Paedophryne* inhabit leaf litter, as seen for most other miniaturized anurans.

## Introduction

Asterophryine frogs represent a large subfamily of the Microhylidae that contains 22 named genera, more than 240 named species, and scores of unnamed forms. The subfamily is monophyletic (Savage 1973; van Bocxlaer et al. 2006; Frost et al. 2006; Roelants et al. 2007; van der Meijden et al. 2007) and centered in the Papuan region of New Guinea and surrounding islands, although one genus (*Oreophryne*) extends as far north as the southern Philippines and another genus (*Cophixalus*) has several species in Queensland, Australia. Member genera of the Asterophryinae vary considerably in morphological and ecological attributes, with burrowing, terrestrial, semi-aquatic, scansorial, and arboreal ecotypes represented (Burton 1986; Menzies 2006; FK, unpubl. data). Sizes vary from 10 to 100 mm snout-vent length (Zweifel 1972; Kraus 2010).

Among those asterophryines of the “terrestrial” ecomorph, which solely inhabit the forest floor, I recently described from southeastern New Guinea a new genus, *Paedophryne*, that included two new species comprising some of the smallest frogs in the world (Kraus 2010). *Paedophryne* was characterized by its small adult size (10.1–11.3 mm in snout-vent length), reduced phalangeal formula, prepollex and prehallux each consisting of a single element, reduced number of presacral vertebrae, *Musculus depressor mandibulae* overlying posterior margin of tympanum, *Musculus adductor mandibularis anterior longus* small and inserting only on lateral portions of frontoparietals, *Musculus submentalis* hypertrophied, and tongue long and straplike. The reduced phalangeal formula imparts a unique appearance to the hands and feet among Papuan frogs, leaving the hands with only three functional digits and the toes with only four. Relationships of the new genus to other asterophryines are the subject of current research, but I earlier gave reasons to expect that *Paedophryne* might be one of the most ancient lineages of asterophryines. The two described species (*Paedophryne kathismaphlox* Kraus, *Paedophryne oyatabu* Kraus) are included among the smallest four or five species of frogs in the world; however, sample sizes of each were limited, making a clearer assessment of size ranking relative to other species uncertain. This is because size differs between sexes in most frog species. Size can be measured relative to a variety of different standards (average or maximum male size, female size, or some combination of them), and many of the world’s smallest frogs have been poorly sampled such that size information is lacking for one sex or another (Lehr and Coloma 2008, table 3).

On a recent survey of an isolated mountain massif in southeastern Papua New Guinea I discovered two more species of *Paedophryne*. Each is of a smaller size than the two species already known in the genus and stands at the lower size limit currently known for anurans (Lehr and Coloma 2008; Kraus 2010) and, therefore, tetrapods (Estrada and Hedges 1996), although both are larger than the world’s smallest fish (Kottelat et al. 2006).

## Materials and methods

All measurements were made with an optical micrometer to the nearest 0.1 mm, except for toe disc width, measured to the nearest 0.03 mm; measurements, terminology, and abbreviations follow Zweifel (1985) and Kraus and Allison (2006): body length from snout−vent (SV); tibia length from heel to outer surface of flexed knee (TL); horizontal diameter of eye (EY); distance from anterior corner of eye to center of naris (EN); internarial distance, between centers of external nares (IN); distance from anterior corner of eye to tip of snout (SN); head width at widest point, typically at the level of the tympana (HW); head length, from tip of snout to posterior margin of tympanum (HL); horizontal tympanum diameter (TY); hand length, from proximal edge of palm to tip of 3^rd^ finger (HandL); foot length, from proximal edge of sole to tip of 4^th^ toe (FootL); width of the fourth toe disc (4thT). I determined sex by presence of vocal slits (males) or examination of gonads (females and males for which the vocal slits were not clearly discernible). Frogs were identified to genus based on diminutive size; presence of eleutherognathine maxillae and a long, strap-like tongue; and the reduced phalangeal pattern that reduces their first digits to mere nubs. The last is unique among Papuan frogs and immediately diagnostic.

I recorded calls in the field using a Sennheiser K6 microphone and a Marantz 660 digital audio recorder; I analyzed call structure using the computer program Avisoft-SASLab Pro(v4.34) available from Avisoft Bioacoustics (http://www.avisoft.com/).

Type specimens are deposited in the Bernice P. Bishop Museum, Honolulu (BPBM) and the Papua New Guinea National Museum and Art Gallery, Port Moresby (PNGNM). All latitude and longitude coordinates use the World Geodetic System, 1984 (WGS 84) and were taken from a hand-held GPS unit.

### Paedophryne dekot

Kraus, sp. n.

urn:lsid:zoobank.org:act:3A6D9296-93A9-45C6-96F4-92FBAE0D428D

http://species-id.net/wiki/Paedophryne_dekot

Figs 1 2A, B

#### Holotype.

BPBM 37753 (field tag FK 15615), alcohol specimen, adult female, collected by F. Kraus and local villagers at Binigun, W slope Mt. Dayman, 9.7071°S, 149.2498°E, 900 m, Milne Bay Province, Papua New Guinea, 1 April 2011.

#### Paratyp.

**(n = 1)***.* BPBM 37754, same data as holotype, except collected 4 April 2011.

#### Diagnosis.

**Figure 1.**
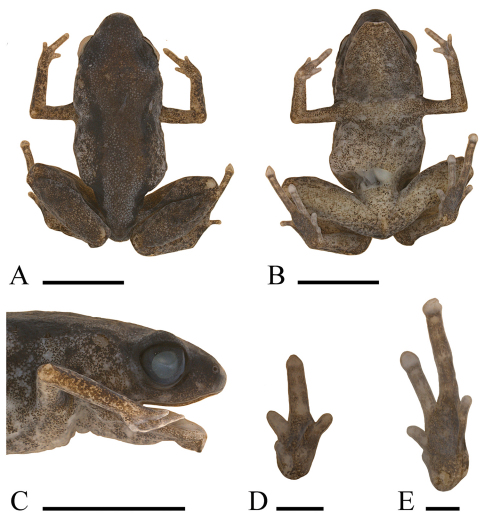
**A** Dorsum, **B** ventrum, **C** side of head, **D** palmar view of left hand, and **E** plantar view of left foot of holotype of *Paedophryne dekot* (BPBM 37753). Scale bars = 5 mm **A–C** and 1 mm **D, E**.

A minute microhylid (female SV = 8.5–9.0 mm) with smooth dorsal skin; a relatively long leg (TL/SV = 0.45–0.46); short and broad snout (EN/SV = 0.067–0.071, IN/SV = 0.106–0.111, EN/IN = 0.60–0.67); relatively large discs on third and fourth toes (4thT/SV = 0.044–0.052, 3rdF/4thT = 0.43–0.58, Fig. 1E); a uniform brown or red-brown dorsum with two large dorsolateral, downward-pointing, black triangular blotches on each side; and a pale gray venter with brown flecks.

#### Comparisons with other species.

*Paedophryne dekot* differs from *Paedophryne kathismaphlox* and *Paedophryne oyatabu*, the only two other species currently described in this genus, in its smaller size (female SV = 8.5–9.0 in *Paedophryne dekot*, 10.4–10.9 mm in *Paedophryne kathismaphlox*, 11.3 mm in *Paedophryne oyatabu*), longer leg (TL/SV = 0.45–0.46 in *Paedophryne dekot*, 0.35–0.39 in *Paedophryne kathismaphlox*, 0.40 in *Paedophryne oyatabu*), shorter snout (IN/SV = 0.106–0.111, EN/IN = 0.60–0.67, EN/SV = 0.067–0.071 in *Paedophryne dekot*; IN/SV = 0.087–0.099, EN/IN = 0.78–0.80 in *Paedophryne kathismaphlox*; EN/SV = 0.062, IN/SV = 0.097 in *Paedophryne oyatabu*), larger discs on third and fourth toes (4thT/SV = 0.044–0.052 in *Paedophryne dekot*, 0.032–0.037 in P*. kathismaphlox*, 0.031 in *Paedophryne oyatabu*; 3rdF/4thT = 0.43–0.58 in *Paedophryne dekot*, 0.66–0.86 in *Paedophryne kathismaphlox*, 0.80 in *Paedophryne oyatabu*), dorsum with two large dorsolateral triangular blotches on each side (dorsum brown vaguely mottled with black or dark brown in *Paedophryne kathismaphlox*, dorsum brown with two dark mid-dorsal chevrons in *Paedophryne oyatabu*), venter pale gray with brown flecks (venter dark brown with scattered light straw-brown or gray flecks in *Paedophryne kathismaphlox* and *Paedophryne oyatabu*), and no brightly colored patch below anus (a burnt-orange patch below anus in *Paedophryne kathismaphlox*).

#### Description of holotype.

An adult female with an incision on right side and across rear of abdomen; liver removed and stored separately for DNA analysis. Head moderately wide (HW/SV = 0.37, Fig. 1A), with steeply oblique loreal region; canthus rostralis rounded, slightly convex when viewed from above; nostrils directed anterolaterally, closer to tip of snout than to eyes; internarial distance much larger than distance from naris to eye (EN/IN = 0.60, IN/SV = 0.111, EN/SV = 0.067); snout rounded when viewed from the side or from above (Fig. 1A, C); eyes moderately large (EY/SV = 0.13; EY/SN = 1.0, Fig. 1C), pupil horizontal; eyelid approximately two-thirds width of interorbital distance; tympanum indistinct and small (TY/SV = 0.044), visible only when skin dries slightly, hidden posterodorsally. Skin smooth; supratympanic fold absent. Fingers unwebbed, flattened; F1 reduced to a vestigial nub; relative lengths 3>2=4>1 (Fig. 1D); discs absent. Subarticular and metacarpal tubercles absent. Toes unwebbed; T3 and T4 with flattened discs and terminal grooves; disc of T4 not wider than penultimate phalanx. Second and fifth toes with round tips and no discs; T1 a vestigial nub; relative lengths of toes 4>3>2=5>1 (Fig. 1E). Subarticular and metatarsal tubercles absent. Plantar and palmar surfaces smooth. Hind legs rather long (TL/SV = 0.46, Fig. 1A). Tongue elongate, straplike, anterior third attached to floor of mouth.

In preservative, dorsum brown with a more-or-less continuous dorsolateral row of black blotchs and flecks; sides and front and rear of thighs pale gray heavily flecked with dark brown. Face dark brown. Ventral surfaces pale gray flecked with dark brown. Iris black.

**Figure 2.**
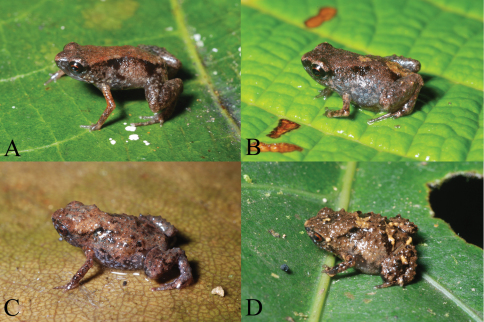
Portraits in life of **A** holotype of *Paedophryne dekot* (BPBM 37753), **B** paratype of *Paedophryne dekot* (BPBM 37754), **C** paratype of *Paedophryne verrucosa* (BPBM 37743), and **D** paratype of *Paedophryne verrucosa* (BPBM 37745).

In life, the holotype was noted as: “Dorsum red brown, sides gray, dorsolateral series of black flecks. Rear of thighs light red brown with black punctations. Face black, posterior to eye spotted with light gray. Venter dark gray with small pale-gray flecks.” The iris was black with a red rim around the pupil, and scattered pale blue-gray flecks are apparent on the lower sides and limbs (Fig. 2A).

*Measurements (in mm).*—SV = 9.0, TL = 4.1, HW = 3.3, HL = 2.9, IN = 1.0, EN = 0.6, SN = 1.2, EY = 1.2, TY = 0.4, 4^th^ T = 0.40.

#### Variation.

There is little mensural difference between the paratype and holotype, except that the former has a somewhat larger tympanum (TY/SV = 0.059) and a slightly longer snout (EN/IN = 0.67). In coloration, the paratype is similar to the holotype but the black dorsal blotching is not concentrated into dorsolateral lines; there is a pale tan blotch over the rump; and the dark brown on the sides, limbs, and ventral surfaces is reduced to even stippling instead of flecking. Liver was also removed from the paratype for DNA analysis. Measurements for the paratype are: SV = 8.5, TL = 3.8, HW = 3.2, HL = 2.7, IN = 0.9, EN = 0.6, SN = 1.1, EY = 1.3, TY = 0.5, 4^th^ T = 0.44.

#### Etymology.

The species name “dekot” is the word for “very small” in Daga, the language spoken in the area from which this species was collected.

#### Range.

**Figure 3.**
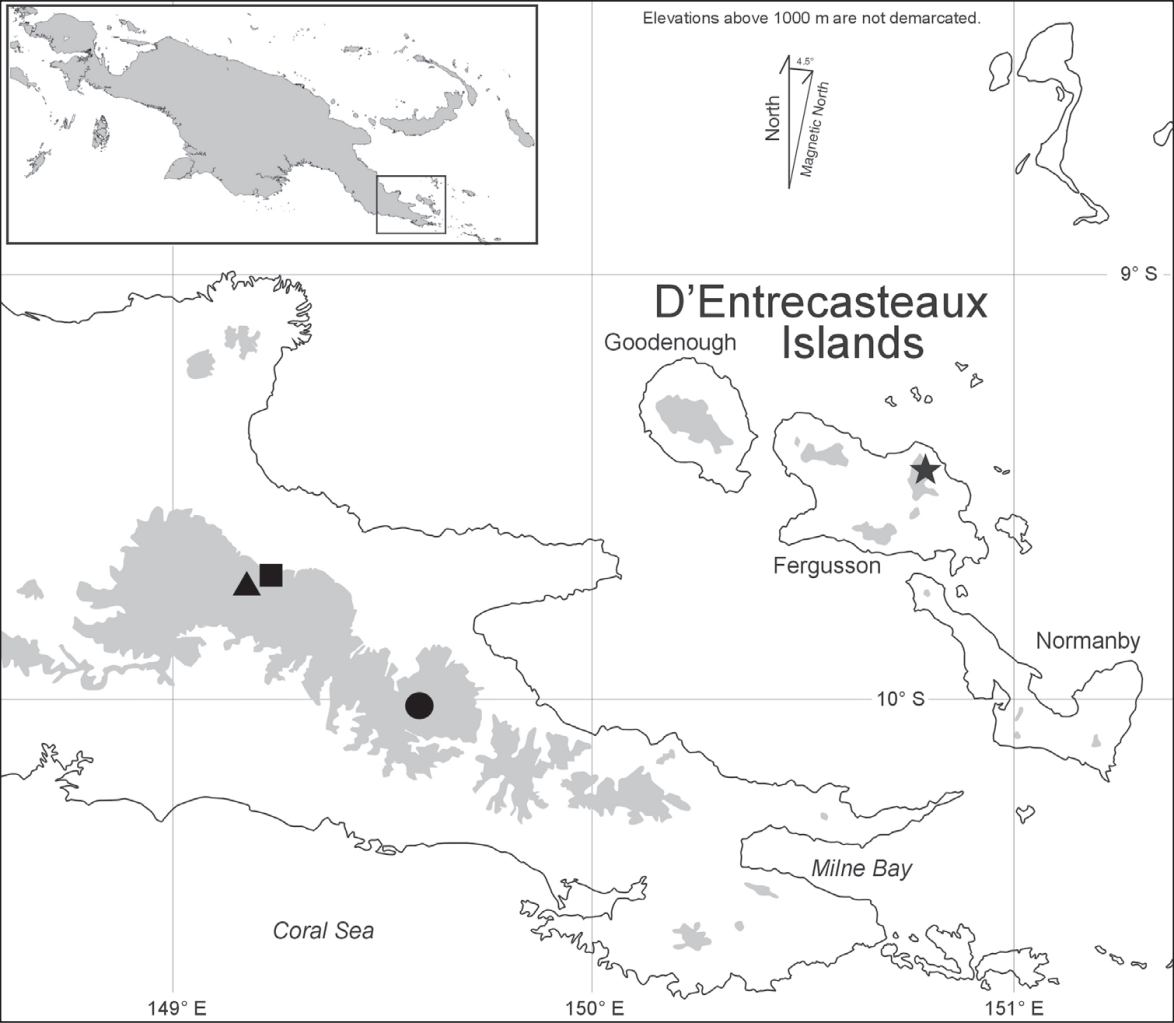
Map of southeastern Papua New Guinea, showing type localities for *Paedophryne dekot* (square) and *Paedophryne verrucosa* (triangle). Only known localities for the related and geographically proximate *Paedophryne kathismaphlox* (filled circle) and *Paedophryne oyatabu* (star) are shown for comparison.

Known only from the western slope of Mt. Dayman in the saddle where it joins Mt. Suckling to the northwest, Milne Bay Province, Papua New Guinea (Fig. 3, square).

#### Ecological notes.

*Paedophryne dekot* inhabits leaf litter on the floor of steeply sloping primary foothill rainforest. Canopy at the type locality was approximately 35 m high; understory was dense, with some moss on trees and ground. This forest type terminates at approximately 1200 m elevation in this area, so *Paedophryne dekot* seems unlikely to occur higher than that.

This species was heard calling from the forest floor in mid- to late afternoon and at dusk but could not be recorded by me.

Both females contained two enlarged, well-yolked, cream-colored eggs and approximately a dozen small white oocytes.

### Paedophryne verrucosa

Kraus, sp. n.

urn:lsid:zoobank.org:act:8DEA8ED4-D3D2-49DC-AFF2-8B8A21CF0044

http://species-id.net/wiki/Paedophryne_verrucosa

Figs 2C, D 4

#### Holotype.

BPBM 37747 (field tag FK 15516), alcohol specimen, adult male, collected by F. Kraus and local villagers at Sota, SE slope Mt. Dayman, 9.7580°S, 149.1822°E, 1860 m, Milne Bay Province, Papua New Guinea, 27 March 2011.

#### Paratype.

**(n = 11)***.* BPBM 37745–46, same data as holotype; BPBM 37743, same data as holotype, except female collected 23 March 2011; BPBM 37744, same data as holotype, except collected 26 March 2011; BPBM 37748–50, PNGNM 24121–22, same data as holotype, except collected 28 March 2011; BPBM 37751–52, same data as holotype, except females collected 29 March 2011.

#### Diagnosis.

A minute microhylid (male SV = 8.1–8.9 mm, female SV = 8.8–9.3 mm) with highly pustulose dorsal skin and plantar surfaces; a relatively short leg (TL/SV = 0.37–0.42); short and broad snout (EN/SV = 0.067–0.080, IN/SV = 0.108–0.123, EN/IN = 0.60–0.70); wide head (HW/SV = 0.38–0.44), fifth toe distinctly shorter than second; relatively large discs on third and fourth toes (4thT/SV = 0.044–0.055); and a light-brown dorsum and sides flecked with black.

#### Comparisons with other species.

*Paedophryne verrucosa* differs from all other members of the genus in its warty plantar surfaces and in having the fifth toe distinctly shorter than the second; it further differs from *Paedophryne kathismaphlox* in its smaller size (male SV = 8.1–8.9 mm in *Paedophryne verrucosa*, 10.1 mm in *Paedophryne kathismaphlox*; female SV = 8.8–9.3 mm in *Paedophryne verrucosa*, 10.4–10.9 mm in *Paedophryne kathismaphlox*), more heavily warty dorsal skin, shorter snout (IN/SV = 0.108–0.123 in *Paedophryne verrucosa*, 0.087–0.099 in *Paedophryne kathismaphlox*; EN/SV = 0.067–0.080 in *Paedophryne verrucosa*, 0.78–0.80 in *Paedophryne kathismaphlox*), larger discs on third and fourth toes (4thT/SV = 0.044–0.055 in *Paedophryne verrucosa*, 0.032–0.037 in *Paedophryne kathismaphlox*), in having the lateral surfaces the same color as the dorsum (lateral surfaces sharply darker than dorsum and punctated with pale gray in *Paedophryne kathismaphlox*), and in generally lacking a colored patch below anus (tan in one specimen of *Paedophryne verrucosa*, burnt-orange patch below anus in all *Paedophryne kathismaphlox*). The new species further differs from *Paedophryne oyatabu* in its smaller size (female SV = 8.8–9.3 mm in *Paedophryne verrucosa*, 11.3 mm in *Paedophryne oyatabu*), heavily warty dorsal skin (smooth in *Paedophryne oyatabu*), shorter snout (EN/SV = 0.067–0.080 in *Paedophryne verrucosa*, 0.062 in *Paedophryne oyatabu*, IN/SV = 0.108–0.123 in *Paedophryne verrucosa*, 0.097 in *Paedophryne oyatabu*), larger discs on third and fourth toes (4thT/SV = 0.044–0.055 in *Paedophryne verrucosa*, 0.031 in *Paedophryne oyatabu*), dorsum brown with black flecks (brown with two darker scapular chevrons in *Paedophryne oyatabu*); it further differs from *Paedophryne dekot* in its heavily warty dorsal skin (smooth in *Paedophryne dekot*), shorter leg (TL/SV = 0.37–0.42 in *Paedophryne verrucosa*, 0.45–0.46 in *Paedophryne dekot*), wider head (HW/SV = 0.38–0.44 in *Paedophryne verrucosa*, 0.37–0.38 in *Paedophryne dekot*), and light-brown dorsum flecked with black (dorsum brown or red-brown with dorsolateral black triangular blotches in *Paedophryne dekot*).

#### Description of holotype.

**Figure 4.**
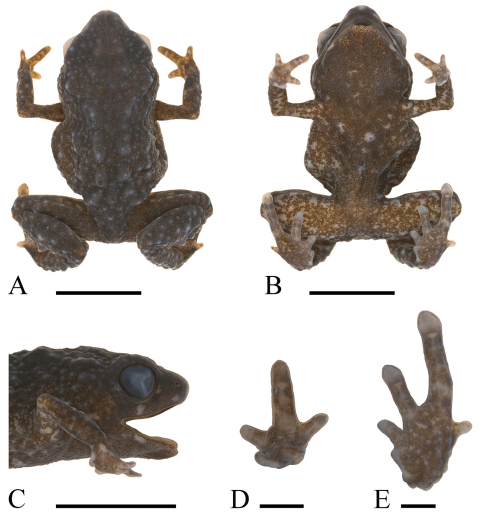
**A** Dorsum, **B** ventrum, **C** side of head, **D** palmar view of left hand, and **E** plantar view of left foot of holotype of *Paedophryne verrucosa* (BPBM 37747). Scale bars = 5 mm **A–C** and 1 mm **D, E**.

An adult male with vocal slits. Head wide (HW/SV = 0.43, Fig. 3A), with steeply oblique loreal region; canthus rostralis rounded, straight when viewed from above; nostrils directed anterolaterally, closer to tip of snout than to eyes; internarial distance much larger than distance from naris to eye (EN/IN = 0.60, IN/SV = 0.112, EN/SV = 0.067); snout somewhat pointed, sharply rounded when viewed from the side or from above (Fig. 4A, C); eyes moderately large (EY/SV = 0.13; EY/SN = 1.0, Fig. 4C), pupil horizontal; eyelid more than half width of interorbital distance; tympanum indistinct and small (TY/SV = 0.056), visible only when skin dries slightly, hidden posterodorsally. Skin granular and highly pustulose dorsally, granular to slightly pustulose ventrally; supratympanic fold absent. Fingers unwebbed, flattened; F1 very reduced in size; relative lengths 3>2=4>1 (Fig. 4D); discs absent. Subarticular and metacarpal tubercles absent; plantar surfaces granular to slightly pustulose. Toes unwebbed; T3 and T4 with flattened discs and terminal grooves; disc of T4 not wider than penultimate phalanx. Second and fifth toes reduced in size, with round tip and no disc; T1 a vestigial nub; relative lengths of toes 4>3>2>5>1 (Fig. 4E). Subarticular and metatarsal tubercles absent, but plantar surfaces heavily pustulose. Hind legs rather short (TL/SV = 0.38, Fig. 4A). Tongue elongate, straplike, anterior one-third attached to floor of mouth.

In preservative, uniform dark brown above, lighter on sides, where many granules are light brown. Face dark brown with few pale-brown spots. Ventral surfaces and front and rear of thighs pale brown heavily flecked with dark brown, the latter most heavily concentrated on chin and throat. Iris black.

*Measurements (in mm).*—SV = 8.9, TL = 3.4, HW = 3.8, HL = 2.9, IN = 1.0, EN = 0.6, SN = 1.2, EY = 1.2, TY = 0.5, 4^th^ T = 0.49.

#### Variation.

Females attain larger size and have smaller tympana than males (Table 1). Mensural variation in the sample is slight, and important differences are not obvious between the sexes.

In preservative, most specimens are somewhat lighter than the holotype, varying to medium brown dorsally, in which case a few small black blotches are evident. Three specimens directly fixed in alcohol each exhibit a pale gray-brown dorsum with a pair of black scapular blotches and a pair of black lumbar blotches, with a smaller mid-dorsal black blotch between the lumbar blotches. This pattern is evident in life in some specimens (Fig. 2D). Ventral coloration can be lighter in overall tone than seen in the holotype due to presence of fewer dark-brown flecks. Both tan and black spots may also occur sparsely in the ventral pattern. Three specimens have a pale-gray patch below the anus.

#### Color in life.

Field notes for paratype BPBM 37743 (Fig. 2C): “Brown with black flecks; warty. Face and venter black with light-gray flecks, posterior of abdomen brown. Rear of thighs brown, each with one large black spot.” For paratype BPBM 37745 (Fig. 2D): “Dark brown with black markings, some tan flecks posteriorly and on legs, tan patch around anus. Venter charcoal gray and light gray.” BPBM 37746 was light brown above with two black scapular triangles and no light anal patch. Its chin, throat, and chest were charcoal gray and its abdomen brown with light-gray punctations and dark-gray flecks. BPBM 37747 was dark brown above and also lacked a light-colored anal patch. Its venter was charcoal gray with light-gray flecks. BPBM 37748 was dorsally as for BPBM 37745 and ventrally as for BPBM 37746 and also lacked an anal patch. PNGNM 24121 was brown and black above and gray flecked with light brown below; PNGNM 24122 tan with black flecks above and gray with light-brown flecks below; BPBM 37749 also brown and black above and light and dark gray below.

#### Call.

**Figure 5.**
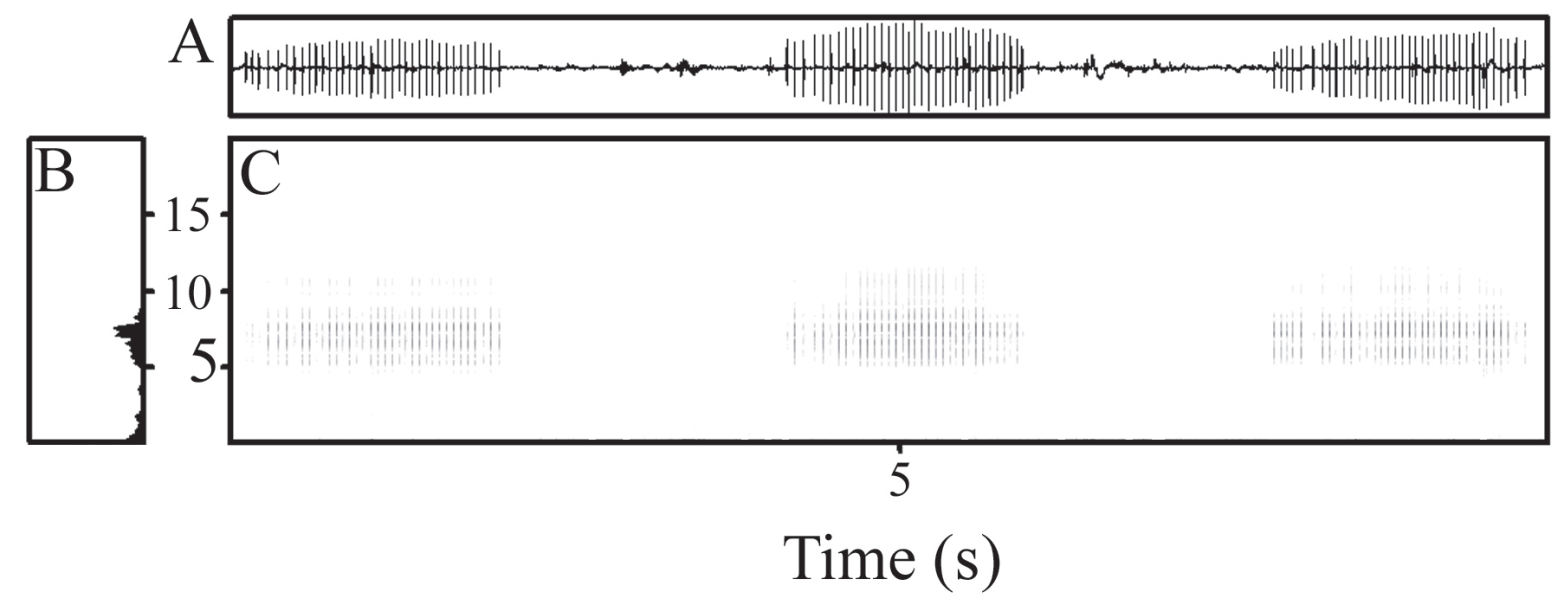
**A** Waveform, **B** power spectrum, and **C** spectrogram (frequency axis in kHz) of three calls of holotype of *Paedophryne verrucosa* (BPBM 37747) recorded on southeastern slope of Mt. Suckling, 27 March 2011, air temperature 14.9°C.

The advertisement call of the holotype was recorded. Each call is a single drawn-out pulsed note given in a long train, with call trains varying from 67–102 s in the two series recorded by me. To the human ear, each call sounds like a quick drag of a finger over a comb. Calls are brief, with average duration varying from 1.210–1.652 s between animals and ranging from 0.712–1.942 s overall (Table 2, Fig. 5). Calls are given at a rate of 0.19–0.46 notes/s, with faster rates at higher temperatures (Table 2). Intervals between calls were longer than the calls themselves and were shorter at higher temperatures, averaging 1.093 s (range 0.897–3.876 s) at 14.9°C, and longer at colder temperatures, averaging 3.694 s (range 1.824–11.655 s) at 19.4°C (Table 2). Calls are highly pulsed, with 15–36 pulses/call, each pulse lasting 0.032–0.066 s (Table 2), and they increase and decrease in maximum amplitude gradually, with maximum amplitude sustained over most of the call duration (Fig. 5A). The power spectrum was rather broad, with a dominant frequency varying from 6510–7890 Hz (Fig. 5B).

#### Etymology.

The species name “verrucosa” is a Latin adjective meaning “full of warts”.

#### Range.

Known only from the southeastern slope of Mt. Suckling near the saddle where it joins Mt. Dayman to the southeast, Milne Bay Province, Papua New Guinea.

#### Ecological notes.

*Paedophryne verrucosa* inhabits leaf litter on the floor of primary mid-montane rainforest, seeming to prefer the lower slopes of steep hillsides. Canopy at the type locality was approximately 35 m high; understory was dense, with fallen trees, *Nastus*, and melastomes common. This mid-montane forest begins at approximately 1200 m elevation, so *Paedophryne verrucosa* seems likely to inhabit forest down to that elevation.

This species typically called at dusk, even continuing through the deafening period of cicada calling at approximately 1800–1830 h, but calling ceased soon after dark. It also frequently called before dawn, and occasional individuals were heard to call briefly in mid-morning. It was not heard by me to call on days lacking rain.

All three females contained two enlarged, well-yolked, cream-colored eggs and approximately a dozen small white oocytes.

**Table 1.** Mensural data for type series of *Paedophryne verrucosa*.

Character	Males (n = 9)		Females (n = 3)	
	mean	range	mean	range
SV (mm)	8.5	8.1–8.9	9.0	8.8–9.3
TL/SV	0.40	0.37–0.42	0.39	0.39–0.40
EN/SV	0.070	0.067–0.074	0.074	0.068–0.080
IN/SV	0.12	0.11–0.12	0.11	0.11–0.11
SN/SV	0.13	0.12–0.14	0.13	0.13–0.14
TY/SV	0.057	0.048–0.062	0.044	0.043–0.045
EY/SV	0.14	0.12–0.14	0.14	0.13–0.15
HW/SV	0.41	0.38–0.44	0.39	0.38–0.40
HL/SV	0.33	0.32–0.35	0.32	0.30–0.33
4thT/SV	0.049	0.045–0.055	0.044	0.044–0.044
EN/IN	0.61	0.60–0.67	0.67	0.60–0.70
HL/HW	0.79	0.76–0.85	0.81	0.80–0.83

**Table 2.** Call data for two specimens of *Paedophryne verrucosa* from Mt. Suckling. Numbers for call parameters are mean (range).

Specimen	Temperature (˚C)	Number of calls	Calling duration (s)	Call rate (calls/s)	Call duration (s)	Interval between calls (s)	Number of pulses/ call	Number of pulses/ call	Pulse length (s)	Dominant frequency (kHz)
BPBM 37747	14.9	13	66.7	0.19	1.652(0.985–1.942)	3.694(1.824–11.655)	30.3(15–36)	30.3(15–36)	0.055(0.051–0.066)	7.27(6.51–7.60)
uncap-tured	19.4	47	102.1	0.46	1.210(0.712–1.315)	1.093(0.897–3.876)	29.8(22–32)	29.8(22–32)	0.040(0.032–0.043)	7.65(7.35–7.89)

## Discussion

These two new frog species are at the lower size limit known for tetrapods and appear to marginally extend that limit. The smallest known tetrapods are all frogs (Lehr and Coloma 2008, Kraus 2010), with the smallest known amniotes being the lizards *Sphaerodactylus ariasae* Hedges and Thomas and *Sphaerodactylus parthenopion* Thomas. These lizards attain snout-vent lengths of 18 mm in both sexes (*Sphaerodactylus ariasae*, Hedges and Thomas 2001) and 16 mm in males and 18 mm in females (*Sphaerodactylus parthenopion*, Schwartz and Henderson 1991). These are approximately the same size as the smallest known salamander, *Thorius arboreus* Hanken and Wake, with average adult size of 17 mm snout-vent length (Hanken and Wake 1994). The only other frog comparable in size to the two new species of *Paedophryne* described herein is *Brachycephalus didactylus* Izecksohn, for which maximum body sizes are slightly larger (9.5 mm in males, 10.7 mm in females, Almeida-Santos et al. 2011). Almeida-Santos et al. (2011) present a considerable range of body sizes for *Brachycephalus didactylus* (6.6–10.7 mm), with mean male sizes of 8.2 and 6.7 mm in two populations and mean female sizes of 10.1 and 8.8 mm in the same populations. However, these authors apparently included juveniles and adults together when deriving their averages, so it is uncertain how well those numbers approximate mean adult body sizes or the range of adult sizes. Furthermore, it is unclear from their study whether body measurements were taken before or after fixation – the former would be expected to introduce greater measurement error because of the malleability of flaccid frogs. In any event, the two new *Paedophryne* species appear to be slightly smaller than *Brachycephalus didactylus*, as measured by maximum body sizes, although the range of male body sizes seen in *Brachycephalus didactylus* appears to widely overlap that seen in the two *Paedophryne* species.

As discussed earlier (Kraus 2010), the useful tabulation of the world’s smallest frogs provided by Lehr and Coloma (2008) overlooked several species from New Guinea, and at least two more species from Borneo also fall in the size range covered by their table (Das and Haas 2010; Matsui 2011). To better reflect body-size information on minute frogs globally, I present in Table 3 these missing records formatted in accord with the presentation provided by Lehr and Coloma (2008). Nineteen species are involved, which represent an increase of almost 50% in the numbers of minute species tabulated by Lehr and Coloma (2008). And I have at hand at least four other minute species of Papuan asterophryines that remain to be described but are omitted from Table 3.

It is uncertain whether the presence of so many minute frogs in the Papuan region represents a biological oddity of that region or whether similar frogs have simply been overlooked or underappreciated elsewhere. Given the difficulty of locating miniaturized frogs in the field and the rate at which they’ve been discovered during the past 15 years, additional miniaturized species no doubt await discovery or description in other poorly surveyed areas of the tropics. For example, further species are known in the diminutive Madagascan *Stumpffia* but have been awaiting description for years (Wollenberg et al. 2008; S.-H. Wu, pers. comm.), and Lehr and Catenazzi (2009) discuss the likelihood that additional miniaturized frogs will be discovered in the Andean cloud forests. Yet, because much attention has been focused over the years on the minute frogs in the genera *Brachycephalus*, *Eleutherodactylus*, and *Sooglossus* (with less attention given to the Madagascan *Stumpffia*), and far more herpetofaunal survey work has been focused on the New World and Asian tropics than on the Papuan and African regions, it seems likely that a disproportionately large complement of overlooked miniaturized frog species is not awaiting discovery in the former regions. However, that remark must be tempered by recognition that diminutive frogs are unlikely to disperse widely, are thus liable to diversification over relatively small areas of mountainous terrain (Lehr and Catenazzi 2009), and, hence, may have hidden diversity in poorly surveyed upland areas of the Neotropics and Southeast Asia.

Given these observations, I tentatively suggest that the remarkable diversity of miniaturized frogs may represent a biological oddity of the Papuan region. Not only are a surprisingly large number of species involved, but the taxonomic diversity is also large: extremely diminutized frogs occur in seven genera of Papuan asterophryines (one genus omitted from Table 3 because its representative species is not yet described), whereas only 12 genera are involved across the remainder of the globe (Lehr and Coloma 2008; Das and Haas 2010). The prevalence of miniaturization in New Guinea may perhaps reflect that open niches were widely available for multiple colonization by early asterophryines ca. 30 MY (van Bocxlaer et al. 2006; Roelants et al. 2007), or the accretionary geological history of New Guinea from formerly isolated island-arc systems (Pigram and Davies 1987; Davies et al. 1996, 1997) may have provided several independent geographic loci for origin of minaturized lineages. Clearer assessment of these options awaits better clarification of asterophryine phylogenetic relationships.

The connection between anuran miniaturization and exploitation of leaf-litter and moss habitats has been briefly discussed previously (Lehr and Coloma 2008; Lehr and Catenazzi 2009; Kraus 2010). The two species under present consideration also fit this pattern, with frogs of both species found active or heard calling only from leaf litter accumulated on the ground. It is worth noting, however, that not all miniaturized frogs are leaf-litter inhabitants: the Papuan *Cophixalus sisyphus* Kraus and Allison and an undescribed *Cophixalus* are arboreal or semi-arboreal (Kraus and Allison 2006, Kraus unpubl. data). Nonetheless, the strong connection with leaf-litter or moss habitats is suggestive that miniaturization is a frequently evolved means to exploit these habitats. Given that anurans with a large range of body sizes reside in leaf litter, it seems likely that the impetus for miniaturization is exploitation of new, minute food resources (e.g., mites) not available to larger frogs (Lehr and Coloma 2008). Given the high surface-area-to-volume ratios of these small frogs, it is hardly surprising that they are restricted to very wet tropical forests.

Also consistent with other diminutive frogs, the new species of *Paedophryne* exhibit clutch sizes at the lower end of those known for anurans. All five females of the two species (2 *Paedophryne dekot*, 3 *Paedophryne verrucosa*) each contained two enlarged ova and a complement of approximately one dozen tiny oocytes. This strongly suggests that clutch size in these species is one or two, although it remains to be determined how frequently females deposit clutches. Other species of minute frogs (e.g., *Brachycephalus didactylus*, *Eleutherodactylus iberia* Estrada and Hedges, *Eleutherodactylus limbatus* Cope, *Eleutherodactylus orientalis* Barbour and Shreve) have even smaller clutches, with females producing only a single egg at a time (Estrada and Hedges 1996). *Brachycephalus didactylus* carries 2–6 mature ova and is surmised to perhaps lay single eggs on different days (Almeida-Santos et al. 2011). Small clutch size in these minute frogs is unsurprising inasmuch as each of these species has non-aquatic oviposition with direct development from eggs into froglets, such direct-developing frog species produce large eggs heavily endowed with yolk, and the minute size of such frogs will constrain the numbers of well-yolked eggs that a female can produce at a single time (Wells 2007). Even if female *Paedophryne* produce several clutches of eggs each year (known for other tropical direct developers, e.g., Townsend and Stewart 1994), these species would still appear to have intrinsically low demographic growth parameters, suggesting that should populations become seriously depleted they would have long recovery times. Nonetheless, from my observations, *Paedophryne verrucosa* at least is very common where it occurs, suggesting that the species may enjoy relatively high survivorship despite the seeming vulnerability conferred by its small body and clutch sizes.

**Table 3.** Body sizes of minute frog species omitted from the survey of Lehr and Coloma (2008) or described since that time. Included are species with body-size tendencies < 16 mm SV. All species are from New Guinea except for *Microhyla* (from Borneo).

Species	Max SV male	Max SV female	Mean SV males	Mean SV females	Mean SV males and females	Range males	Range females	Sample size males	Sample size females	References
Paedophryne dekot	–	9.0	–	8.8	–	–	8.5–9.0	–	2	this study
Paedophryne verrucosa	8.9	9.3	8.5	9.0	8.7	8.1–8.9	8.8–9.3	9	3	this study
Paedophryne kathismaphlox	10.1	10.9	–	10.6	10.5	–	10.4–10.9	1	3	Kraus 2010
Paedophryne oyatabu	–	11.3	–	–	–	–	–	–	1	Kraus 2010
Microhyla perparva	10.5	12.4	–	–	–	10.1–10.5	11.4–12.4	–	–	Matsui 2011
Oreophryne minuta	11.5	–	–	–	–	9.2–11.5	–	4	–	Richards and Iskandar 2000
Choerophryne allisoni	11.6	–	11.6	–	–	11.5–11.6	–	2	–	Richards and Burton 2003
Aphantophryne minuta	–	11.8	–	–	–	–	–	–	1	Zweifel and Parker 1989
Choerophryne burtoni	12.4	–	12.3	–	–	12.1–12.4	–	3	–	Richards et al. 2007
Microhyla borneensis	12.8	18.8	11.7	18.4	–	10.6–12.8	17.9–18.8	8	2	Das and Haas 2010
Cophixalus kethuk	13.5	15.0	12.9	13.9	13.4	12.4–13.5	13.2–15.0	6	5	Kraus and Allison 2009
Cophixalus sisyphus	14.1	13.6	13.3	13.6	13.4	12.0–14.1	13.5–13.6	24	2	Kraus and Allison 2006
Cophixalus linnaeus	14.7	16.7	14.1	15.6	14.7	13.4–14.7	14.9–16.7	4	3	Kraus and Allison 2009
Choerophryne arndtorum	14.8	–	13.8	–	–	11.2–14.8	–	13	–	Günther 2008
Choerophryne amomani	15.1	–	13.8	–	–	11.8–15.1	–	9	–	Günther 2008
Austrochaperina minutissima	15.8	16.6	15.5	–	15.7	15.0–15.8	–	4	1	Günther 2009
Cophixalus iovaorum	16.0	17.2	14.5	16.9	14.6	13.2–16.0	16.6–17.2	29	2	Kraus and Allison 2009
Cophixalus tomaiodactylus	16.1	16.6	14.2	15.7	14.7	13.2–16.1	14.2–16.6	11	5	Kraus and Allison 2009
Cophixalus desticans	16.2	19.1	14.6	18.4	14.8	13.1–16.2	17.6–19.1	32	2	Kraus and Allison 2009

## Supplementary Material

XML Treatment for Paedophryne dekot

XML Treatment for Paedophryne verrucosa

## References

[B1] Almeida-Santos M Siqueira CC van Sluys M Rocha CFD (2011) Ecology of the Brazilian Flea Frog *Brachycephalus didactylus* (Terrarana: Brachycephalidae). Journal of Herpetology 45: 251-255. 10.1670/10-015.1

[B2] Burton TC (1986) A reassessment of the Papuan subfamily Asterophryinae (Anura: Microhylidae). Records of the South Australia Museum 19: 405-450.

[B3] Das I Haas A (2010) New species of *Microhyla* from Sarawak: Old World’s smallest frogs crawl out of miniature pitcher plants on Borneo (Amphibia: Anura: Microhylidae). Zootaxa 2571: 37-52.

[B4] Davies H Perembo R Winn R KenGemar R (1997) Terranes of the New Guinea Orogen. In: Hancock G (Ed) Proceedings of the Geology Exploration and Mining Conference.: 61-66.

[B5] Davies H Winn R KenGemar P (1996) Evolution of the Papuan Basin–a view from the orogen. In: Buchanan P (Ed). Petroleum exploration, development and production in Papua New Guinea. PNG Chamber of Mines and Petroleum, Port Moresby, Papua New Guinea: 53-62.

[B6] Estrada AR Hedges SB (1996) At the lower size limit in tetrapods: a new diminutive frog from Cuba (Leptodactylidae: *Eleutherodactylus*). Copeia 1996: 852-859. 10.2307/1447647

[B7] Frost DR Grant T Faivovich J Bain RH Haas A Haddad CFB de Sá RO Channing A Wilkinson M Donnellan SC Raxworthy CJ Campbell JA Blotto BL Moler P Drewes RC Nussbaum RA Lynch JD Green DM Wheeler WC (2006) The amphibian tree of life. Bulletin of the American Museum of Natural History 297: 1–370. Available online at: http://digitallibrary.amnh.org/dspace/handle/2246/5781

[B8] Günther R (2008) Descriptions of four new species of *Choerophryne* (Anura, Microhylidae) from Papua Province, Indonesian New Guinea. Acta Zoologica Sinica 54: 653-674.

[B9] Günther R (2009) A new and minute species of *Austrochaperina* (Amphibia: Anura: Microhylidae) from western New Guinea. Vertebrate Zoology 59: 81-89.

[B10] Hanken J Wake DB (1994) Five new species of minute salamanders, genus *Thorius* (Caudata: Plethodontidae), from northern Oaxaca, Mexico. Copiea 1994: 573-590. 10.2307/1447174

[B11] Hedges SB Thomas R (2001) At the lower size limit in amniote vertebrates: a new diminutive lizard from the West Indies. Caribbean Journal of Science 37: 168–173. Available online at: http://academic.uprm.edu/publications/cjs/Vol37b/37_168–173.pdf

[B12] Kottelat M Britz R Hui TH Witt K-E (2006) *Paedocypris*, a new genus of Southeast Asian cyprinid fish with a remarkable sexual size dimorphism, comprises the world’s smallest vertebrate. Proceedings of the Royal Society B 273: 895-899. 10.1098/rspb.2005.3419 16627273 PMC1560243

[B13] Kraus F (2010) New genus of diminutive microhylid frogs from Papua New Guinea. Zookeys 48: 39–59. Available online at: http://www.pensoft.net/J_FILES/1/articles/446/446-G-1-layout.pdf

[B14] Kraus F Allison A (2006) Three new species of *Cophixalus* (Anura: Microhylidae) from southeastern New Guinea. Herpetologica 62: 202-220. 10.1655/05-09.1

[B15] Kraus F Allison A (2009) New species of *Cophixalus* (Anura: Microhylidae) from Papua New Guinea. Zootaxa 2128: 1-38.

[B16] Lehr E Catenazzi A (2009) A new species of minute *Noblella* (Anura: Strabomantidae) from southern Peru: the smallest frog of the Andes. Copeia 2009: 148-156. 10.1643/CH-07-270

[B17] Lehr E Coloma LA (2008) A minute new Ecuadorian Andean frog (Anura Strabomantidae, *Pristimantis*). Herpetologica 64: 354-367. 10.1655/07-089.1

[B18] Matsui M (2011) Taxonomic revision of one of the Old World’s smallest frogs, with description of a new Bornean *Microhyla* (Amphibia, Microhylidae). Zootaxa 2814: 33-49.

[B19] Menzies JI (2006) The Frogs of New Guinea and the Solomon Islands. Pensoft, Sofia, Bulgaria, 346 pp.

[B20] Pigram C Davies H (1987) Terranes and the accretion history of the New Guinea Orogen. BMR Journal of Australian Geology and Geophysics 10: 193-211.

[B21] Richards SJ Burton TC (2003) A new species of *Choerophyrne* (Anura: Microhylidae) from Southern Highlands Province, Papua New Guinea. Transactions of the Royal Society of South Australia 127: 47-51.

[B22] Richards SJ Iskandar D (2000) A new minute *Oreophryne* (Anura: Microhylidae) from the mountains of Irian Jaya, Indonesia. Raffles Bulletin of Zoology 48: 257-262.

[B23] Richards SJ Dahl CS Hiaso J (2007) Another new species of *Choerophryne* (Anura: Microhylidae) from Southern Highlands Province, Papua New Guinea. Transactions of the Royal Society of South Australia 131: 135-141.

[B24] Roelants K Gower DJ Wilkinson M Loader SP Biju SD Guillaume K Moriau L Bossuyt F (2007) Global patterns of diversification in the history of modern amphibians. Proceedings of the National Academy of Sciences of the United States of America 104: 887–892. 10.1073/pnas.0608378104 PMC1783409 17213318

[B25] Savage J (1973) The geographic distribution of frogs: patterns and predictions. In: Vial J (Ed). Evolutionary Biology of the Anurans. University of Missouri Press, Columbia: 351-445.

[B26] Schwartz A Henderson RW (1991) Amphibians and Reptiles of the West Indies: Descriptions, Distributions, and Natural History. University of Florida Press, Gainesville, Florida, 720 pp.

[B27] Townsend DS Stewart MM (1994) Reproductive ecology of the Puerto Rican frog *Eleutherodactylus coqui*. Journal of Herpetology 28: 34-40. 10.2307/1564677

[B28] van Bocxlaer I Roelants K Biju S Nagaraju J Bossuyt F (2006) Late cretaceous vicariance in gondwanan amphibians. PLoS ONE, e74. 10.1371/journal.pone.0000074 PMC1762348 17183706

[B29] van der Meijden A Vences M Hoegg S Boistel R Channing A Meyer A (2007) Nuclear gene phylogeny of narrow-mouthed toads (family: Microhylidae) and a discussion of competing hypotheses concerning biogeographical origins. Molecular Phylogenetics and Evolution 44: 1017–1030. doi: 10.1016/j.ympev.2007.02.008 10.1016/j.ympev.2007.02.008 17369057

[B30] Wells KD (2007) The Ecology and Behavior of Amphibians. University of Chicago Press, Chicago, Illinois, 1148 pp.

[B31] Wollenberg KC Vieites DR van der Meijden A Glaw F Cannatella DC Vences M (2008) Patterns of endemism and species richness in Malagasy cophyline frogs support a key role of mountainous areas for speciation. Evolution 62: 1890-1907. 10.1111/j.1558-5646.2008.00420.x 18485110

[B32] Zweifel RG (1972) A revision of the frogs of the subfamily Asterophryinae family Microhylidae. Bulletin of the American Museum of Natural History 148:411–546. [http://digitallibrary.amnh.org/dspace/handle/2246/1102]

[B33] Zweifel RG (1985) Australian frogs of the family Microhylidae. Bulletin of the American Museum of Natural History 182: 265–388.[http://digitallibrary.amnh.org/dspace/handle/2246/578]

[B34] Zweifel RG Parker F (1989) New species of microhylid frogs from the Owen Stanley Mountains of Papua New Guinea and resurrection of the genus *Aphantophryne*. American Museum Novitates 2954: 1–20. [http://digitallibrary.amnh.org/dspace/handle/2246/5109]

